# Ozonolysis of Terpene Flavor Additives in Vaping Emissions: Elevated Production of Reactive Oxygen Species and Oxidative Stress

**DOI:** 10.1021/acs.chemrestox.4c00051

**Published:** 2024-05-22

**Authors:** Wonsik Woo, Linhui Tian, Michael Lum, Alexa Canchola, Kunpeng Chen, Ying-Hsuan Lin

**Affiliations:** † Environmental Toxicology Graduate Program, University of California, Riverside, California 92521, United States; ‡ Department of Environmental Sciences, University of California, Riverside, California 92521, United States

## Abstract

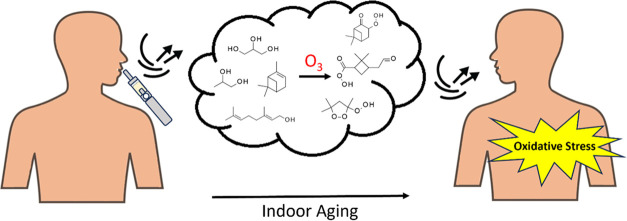

The production of e-cigarette aerosols through vaping processes is known to cause the formation of various free radicals and reactive oxygen species (ROS). Despite the well-known oxidative potential and cytotoxicity of fresh vaping emissions, the effects of chemical aging on exhaled vaping aerosols by indoor atmospheric oxidants are yet to be elucidated. Terpenes are commonly found in e-liquids as flavor additives. In the presence of indoor ozone (O_3_), e-cigarette aerosols that contain terpene flavorings can undergo chemical transformations, further producing ROS and reactive carbonyl species. Here, we simulated the aging process of the e-cigarette emissions in a 2 m^3^ FEP film chamber with 100 ppbv of O_3_ exposure for an hour. The aged vaping aerosols, along with fresh aerosols, were collected to detect the presence of ROS. The aged particles exhibited 2- to 11-fold greater oxidative potential, and further analysis showed that these particles formed a greater number of radicals in aqueous conditions. The aging process induced the formation of various alkyl hydroperoxides (ROOH), and through iodometric quantification, we saw that our aged vaping particles contained significantly greater amounts of these hydroperoxides than their fresh counterparts. Bronchial epithelial cells exposed to aged vaping aerosols exhibited an upregulation of the oxidative stress genes, *HMOX-1* and *GSTP1*, indicating the potential for inhalation toxicity. This work highlights the indirect danger of vaping in environments with high ground-level O_3_, which can chemically transform e-cigarette aerosols into new particles that can induce greater oxidative damage than fresh e-cigarette aerosols. Given that the toxicological characteristics of e-cigarettes are mainly associated with the inhalation of fresh aerosols in current studies, our work may provide a perspective that characterizes vaping exposure under secondhand or thirdhand conditions as a significant health risk.

## 1 Introduction

E-cigarettes have largely been considered as a harm-reduction device compared to conventional tobacco cigarettes, and their use has been increasing since their conception.^1,2^ E-cigarettes are devices that heat and aerosolize e-liquids into a gaseous and particle suspension that mimics the process of smoking when inhaled.^2^ E-liquids are usually composed of propylene glycol (PG), glycerol (VG), various flavoring compounds, and nicotine. Currently, there are hundreds of different compounds that are used as flavoring agents in e-liquids,^3^ and a common class of these compounds is terpenes.^4^ Terpenes are unsaturated hydrocarbons that are characterized by their pungent aromas, which are responsible for the scents of many different plant species.^4^ On the other hand, terpenes are biogenic precursors for secondary organic aerosol (SOA) formation when they react with atmospheric oxidants, and inhalation of these oxygenated aerosols has been shown to induce a variety of respiratory and systemic pathologies.^5−7,56^

Like conventional cigarettes, there is also a risk of secondhand exposure to vaping. Vaping indoors has negative effects on air quality,^8−10^ and it has previously been reported that indoor areas with active vaping have PM_2.5_ concentrations that can reach up to 1500 μg m^–3^ for extended periods of time.^8^ Once in the air, vaping emissions can be inhaled by bystanders, potentially being a risk for sensitive populations, such as people who already have compromised respiratory systems.^11^ Vaping emissions also tend to linger and stay suspended within the air,^11,12^ which presents the possibility for their chemical aging by ambient oxidants found in indoor air.^13^ If terpenes are present in these vaping emissions, that suggests the likelihood of an O_3_-mediated aging process that will chemically transform the original vaping emissions into new particles that contain more oxygenated compounds.^14−16^ Various cytotoxic products are formed from the ozonolysis of terpenes, such as carbonyls,^17^ and these compounds exert their toxic effects through a multitude of mechanisms.^18−20^ However, it is also known that this process produces organic hydroperoxides that are relatively stable in the particle phase,^21^ and exposing human bronchial epithelial cells to various terpene SOAs resulted in indications of ROS exposure and the development of oxidative stress.^5−7,22^ Therefore, it is likely that vaping emissions aged by O_3_ have the potential to be much more harmful than their fresh counterparts.

In this work, we compare the oxidative potential of freshly generated vaping aerosols and aerosols that have undergone O_3_ aging. To achieve this, we conducted chamber studies under conditions that reflect the aging process of vaping emissions in indoor environments. Vaping aerosols were collected onto filters, and various online and offline analytical techniques were employed to characterize the chemical composition and oxidative potential of these aerosols. Overall, the findings of this study may provide a perspective that characterizes secondhand or thirdhand vaping aerosol exposure as a significant respiratory health risk as well as insight into the potential mechanisms that facilitate the toxicity of these aged aerosols.

## 2 Materials and Methods

### 2.1 Chemicals

1,2-Propanediol (PG), sodium hydroxide in water (NaOH, 1M), and *tert*-butyl hydroperoxide solution (TBHP, 70% in H_2_O) were purchased from Tokyo Chemical Industry. Glycerol (VG), dimethyl sulfoxide (DMSO), and KCl/HCl buffer solution (pH 2.00) were purchased from Fisher Chemical. Geraniol (99%) was purchased from Acros Organics, and α-pinene (>99%) was purchased from Sigma-Aldrich. 5-*tert*-Butoxycarbonyl-5-methyl-1-pyrroline-*N*-oxide (BMPO) and 2′-7′-dichlorodihydrofluorescein diacetate (DCFH_2_DA) were purchased from Cayman Chemical Company. Phosphate-buffered saline (1×) (PBS) was purchased from Corning. Potassium iodide (KI, 99%) was purchased from Thermo Scientific.

### 2.2 E-Liquid Mixtures

A mixture of 30% propylene glycol (PG) and 70% vegetable glycerin (VG) was utilized as the diluent in our e-liquids. Terpenes, such as α-pinene and geraniol, were added to the PG/VG mixture to create a 3% terpene mixture. A 3% mixture of GG#4 (Gold Coast Terpenes), a commercial terpene mixture, was prepared in the same manner. GG#4 consisted of α-pinene, α-terpinene, terpineol, limonene, β-pinene, 3-carene, linalool, terpinolene, ocimene, geraniol, β-caryophyllene, α-humulene, camphene, α-phellandrene, α-cedrene, p-cymene, myrcene, nerolidol, and pulegone.

### 2.3 E-Cigarette Device

The e-cigarette device was composed of a battery (Silo, CCELL) that operated at 3.6 V and a 510-thread cartridge with a coil resistance of 1.4 Ω (CCELL). Before each chamber experiment, the cartridge was filled with 450 μL of our e-liquid mixtures and preconditioned by taking five puffs before injection into the chamber.

### 2.4 Chamber Experiments to Simulate O_3_ Aging

In urban areas that are heavily polluted with anthropogenic pollutants, there is an observed increase in ground-level O_3_ concentrations. In these environments, O_3_ concentrations can regularly reach or exceed 100 ppbv.^23,24^ Chamber experiments with controlled levels of O_3_ were done to simulate the aging process of e-cigarette emissions under these high O_3_ conditions. For our chamber experiments, we utilized a 2 m^3^ fluorinated ethylene propylene smog chamber. Prior to each experiment, we flushed the chamber overnight with zero air at a flow rate of 30 LPM to remove any potential contaminants. For fresh conditions, the chamber was filled with zero air, and 30 puffs of vaping emissions were injected into the chamber using an e-cigarette puffing machine (CSM-eSTEP, CH Technologies). The puffing topography followed the CORESTA recommended protocol with a puff period of 3 s, a puff interval of 30 s, and a puff volume of 55 mL.^25^ The aerosols were collected directly after injection onto 25 and 47 mm PTFE filters (Zefluor, Pall Laboratory, 1 μm pore size). For the aged conditions, the chamber was filled with 100 ppbv of O_3_ with a laboratory benchtop O_3_ generator (A2Z Ozone 3 GLAB) utilizing pure O_2_. Real-time measurements of O_3_ concentration were taken with a photometric O_3_ analyzer (Advanced Pollution Instrumentation, model 400A). After injecting 30 puffs, the vaping emissions were allowed to undergo ozonolysis and age for 1 h before filter collection. To mitigate the potential loss of volatile compounds from the filters, they were immediately stored at −20 °C before further analysis. To minimize the time the filters spent at room temperature, they were extracted within 30 min after they had been taken out of −20 °C. A scanning electrical mobility spectrometer (SEMS, Brechtel) was employed to monitor particle number and volume concentrations with a scan range of 10–800 nm and 140 size bins. Assuming a particle density of 1.0 g cm^–3^, the mass loading on each filter was calculated using the recorded particle concentrations by multiplying the chamber volume by the average mass concentration during the sampling period (Figure S1).

### 2.5 Electron Paramagnetic Resonance (EPR) Spin-Trapping

Aerosol samples were collected onto filters at a flow rate of 20 LPM to reach a mass loading of around 1 mg. These filters were stored in a 500 μL microcentrifuge tube (Fischer Scientific) and stored at −20 °C before analysis. A 20 mM solution of 5-*tert*-butoxycarbonyl-5-methyl-1-pyrroline-*N*-oxide (BMPO) was prepared before analysis by dissolving 2 mg of BMPO into 500 μL of Milli-Q water. The filters were extracted with 150 μL of BMPO solution by vigorously vortexing for 12 min. The extract was then collected into a 50 μL capillary tube and placed into the sample cavity of an ESR5000 (Magnettech). EPR spectra were acquired with a sweep range of 333–340.5 mT, a sweep time of 22 s, a modulation amplitude of 0.1 mT, a modulation frequency of 100 kHz, and a microwave power of 20 mW. A digital RC filter of 0.08 s was used in postprocessing. Each spectrum was an average of 25 accumulations to achieve an optimal S/N ratio. EPR spectra underwent simulation with the MATLAB package, EasySpin. The “garlic” function was chosen for spectral simulation, and hyperfine parameters were adopted from Fang et al.^26^

### 2.6 Oxidative Potential Assay

The oxidative potential of our fresh and aged vaping particles was determined by monitoring the rate of 2′,7′-dichlorodihydrofluorescein (DCFH_2_) oxidation into the fluorescent 2′-7′-dichlorofluorescein (DCF). The preparation of DCFH_2_ was adopted from a previous work done by Canchola et al.^19^ Filters were extracted in the dark with a corresponding volume of DCFH_2_ solution to create a final concentration of 3 mg mL^–1^ of vaping particles. The same extraction method as described in Section 2.5 was used, where the filter sample was vortexed for 12 min. After extraction, 100 μL of the sample was pipetted into a 96-well, black, clear bottom plate (Corning), and its fluorescence was measured with a plate reader (SpectraMax iD5) with an excitation wavelength of 485 nm and an emission wavelength of 535 nm. Each condition had 3 replicates for statistical analysis. A negative control of 3 filter blanks was utilized, and the oxidative potential of the samples was expressed as fold change relative to the negative control.

### 2.7 Iodometry

To determine the concentration of hydroperoxides present in our samples, we utilized iodometry. A 60 mM solution of KI was prepared by dissolving KI in a pH 2 KCl/HCl buffer solution. Filters were extracted with a corresponding volume of KI solution to create a final concentration of 3 mg mL^–1^. The same extraction method as described in Section 2.5 was used, where the filter sample was vortexed for 12 min. The aged samples were diluted 10-fold after extraction with the prepared KI solution. After extraction, the mixture was allowed to react in the dark at room temperature for an hour. After 1 h, 100 μL of the sample was pipetted into a 96-well, flat, clear bottom plate (Greiner Bio-One), and its absorbance at 350 nm was measured with a plate reader (SpectraMax iD5). Each condition had 3 replicates for statistical analysis. A calibration curve of 1 μM to 1 mM of *tert*-butyl hydroperoxide (TBHP) was made for each plate, and it was utilized to calculate the concentration of total peroxides in our samples (Figure S2). TBHP′s reactivity to I^–^ in the iodometry reaction is not fully representative of the peroxides that are formed during the aging process and should not be used for the absolute quantification of unknown organic peroxides with differing reactivities. Therefore, our data was expressed as TBHP equivalences as a measure of relative abundance.

### 2.8 FIGAERO-ToF-CIMS

The molecular formulas of aerosol-phase vaping emission constituents were measured offline using an iodide-adduct time-of-flight chemical ionization mass spectrometer coupled with a Filter Inlet for Gases and AEROsols system (FIGAERO-ToF-CIMS, Aerodyne Research Inc.). The data were analyzed using Tofware v3.2.5 coupled with the Igor Pro 7.0.8 (WaveMetrics) environment.^27^ The temperature parameters for thermal desorption were as follows: (1) ramping from ∼20 to 200 °C in 20 min; (2) 15 min soaking period at 200 °C; (3) cooling from 200 to 25 °C in 10 min.

### 2.9 Cell Culture and Exposure

Human bronchial epithelial cells (BEAS-2B) were purchased from the American Type Culture Collection (ATCC). Cells were cultured in 75 cm^2^ tissue culture flasks (Fisher Scientific) with LHC-9 medium (Gibco). Cells were incubated at 37 °C and 5% CO_2_. The growth media were replaced every 2–3 days, and the cells were subpassaged once 70–80% confluent. Once confluent, the cells were transferred onto 48-well plates (Corning) with a seeding density of 10^5^ cells per well. The cells were allowed 24 h for attachment before vaping aerosol exposures. Once seeded onto 48-well plates, BEAS-2B cells were exposed to 0.3 and 3 mg mL^–1^ of fresh and aged GG#4 vaping particles. The aerosols were collected onto 47 mm PTFE filters at a flow rate of 20 LPM to obtain a mass loading of 4–5 mg. The filters were then extracted by vortexing with cell media to achieve a concentration of 3 mg mL^–1^. Once exposed, the cells were left to incubate at 37 °C and 5% CO_2_ for 6 h. Each condition had 4 replicates, and untreated cells were used as a negative control. After 6 h, the cells were lysed with 200 μL of TRI Reagent (Zymo Research), and RNA was extracted using a Direct-Zol RNA MiniPrep Kit (Zymo Research). A Nanodrop ND-2000C was used to measure the concentration of the extracted RNA as well as its A260:280 ratios. All samples had A260:280 ratios that were within 1.8–2.0. The extracted RNA was stored at −80 °C until further analysis.

### 2.10 Gene Expression Analysis

The expression of *HMOX-1* and *GSTP1* mRNA was measured with a QuantiNova SYBR Green RT-PCR kit (Qiagen) and QuantiTect Primer Assays for *HMOX-1* (GeneGlobe ID: QT00092645) and *GSTP1* (GeneGlobe ID: QT00086401). RT-qPCR was run on a CFX96 (Bio-Rad). Reverse transcription took place for 10 min at 50 °C, and 2 min at 95 °C was required for initial heat activation. The qPCR cycling protocol involved 5 s at 95 °C and 10 s at 60 °C for 40 cycles. Gene expression was analyzed by the 2^–ΔΔ-Ct^ method, normalized to a housekeeping gene, *ACTB* (GeneGlobe ID: QT01680476), and expressed as fold change or log_2_ fold change relative to expression in the negative control.

### 2.11 Data Analysis

GraphPad Prism 10 was utilized for the statistical analysis of our oxidative potential, iodometry, and gene expression data. Statistical analysis involved the usage of two-way ANOVA along with Tukey’s HSD post hoc test.

## 3 Results and Discussion

### 3.1 Increased Oxidative Potential of Aged Vaping Aerosols

**Figure 1:**
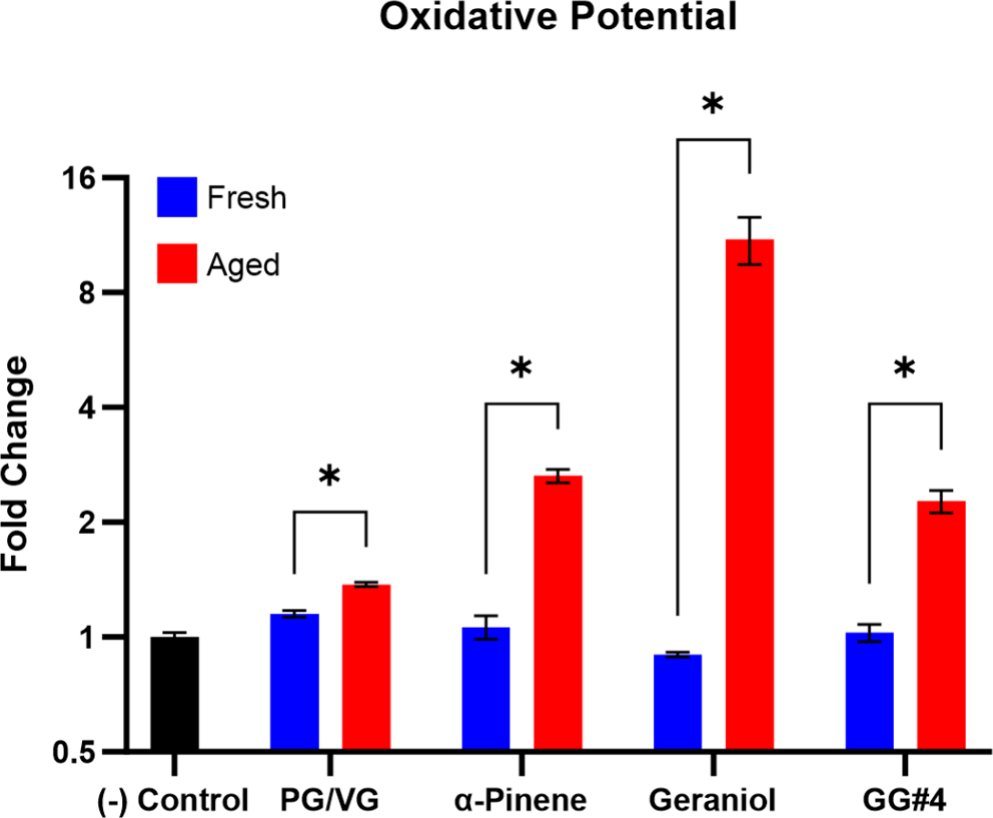
Oxidative potential of fresh and aged vaping particles determined by the oxidation of DCFH_2_ to the fluorescent DCF. The data is expressed as the mean fold change of 3 filter replicates (*n* = 3) relative to the negative control ± the standard error of the mean (SEM). Two-way ANOVA was used to determine statistical significance and * indicates *P* < 0.05.

We assessed the relative abundance of particle-bound ROS in e-cigarette aerosols by monitoring the oxidation of our oxidant probe, DCFH_2_. As shown in Figure 1, the aged vaping particles had greater oxidizing capabilities, which resulted in a greater fluorescent intensity. This indicates greater oxidative potential compared to their fresh counterparts. Since the DCFH_2_ assay is nonspecific for what oxidants can influence the results,^28,29^ we are not able to determine what oxidizing species are responsible for the observed increase in oxidative potential when using conventional assays. The common forms of ROS include OH, HO_2,_ and O_2_^–^ radicals, as well as nonradical species such as hydrogen peroxide and quinones.^29^ It is unlikely for there to be a significant amount of unreacted particle-bound radicals in our system, but it is reasonable for there to be an abundance of peroxides. The formation of hydroperoxides from the ozonolysis of terpenes is a well-established pathway, where reactions of Criegee intermediates with volatile organic compounds (VOCs) containing alcohol and carboxyl functional groups form multifunctional hydroperoxides that are prone to aqueous decomposition.^30−32^ Therefore, it is reasonable to hypothesize that the increased oxidative potential can be explained by the formation of hydroperoxides from the ozonolysis of terpene flavor additives. The greater oxidative ability of our aged vaping particles is also likely to be accompanied by an elevated probability of inducing oxidative damage to the respiratory epithelium of people who experience secondhand exposure.^5^ These particles could directly induce deleterious phenotypes such as lipid peroxidation, DNA lesions, and mitochondrial dysfunction.^33,34^ The aerodynamic diameter of our vaping particles was measured to be in the submicrometer range (Figure S3), which allows for efficient particle deposition within the bronchiolar and alveolar regions of the respiratory tract.^35^ Accompanying direct oxidative damage to the respiratory epithelium, there may be diffusion of these vaping particles and their dissolved constituents into the systemic circulation, leading to potential endothelial and cardiovascular pathologies.^35,56^ This effect could potentially be more severe in people who already have compromised respiratory systems, such as those with asthma and chronic obstructive pulmonary disease (COPD).^35,36^ Individuals with these pre-existing conditions are subject to an increased risk of experiencing respiratory damage from exposure to particulate matter, and there is evidence that suggests these individuals possess a lower exposure threshold for experiencing particulate matter-induced toxicity.^36^

### 3.2 Formation of Radicals from Particle–Water Interactions

**Figure 2:**
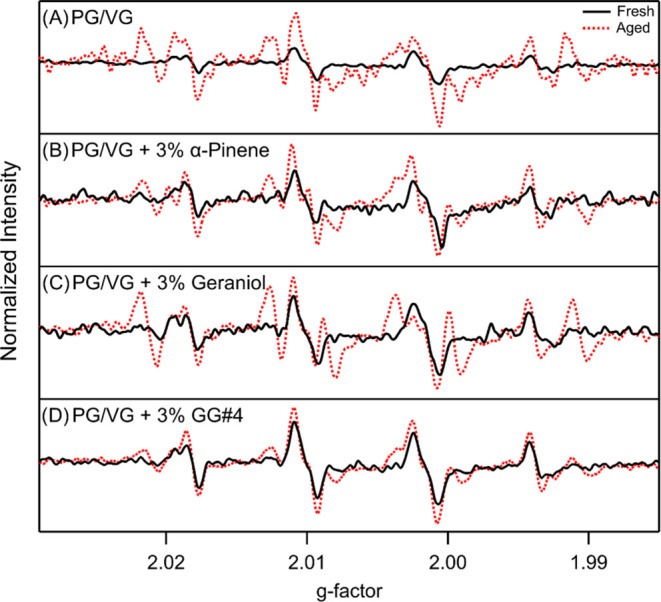
Comparison of radical abundance resulting from the aqueous decomposition of fresh and aged vaping particles: (A) PG/VG, (B) PG/VG + 3% α-pinene, (C) PG/VG + 3% geraniol, and (D) PG/VG + 3% GG#4. Spectra are observed signals whose intensities were normalized by their mass loading on their respective filters.

The epithelial lining of the respiratory tract is surrounded by a thick layer of mucus that acts as a barrier against external insults such as airborne particulate matter. This mucus layer is mainly composed of water, salts, and various mucin proteins secreted by the residing club and goblet cells.^37^ Once inhaled, vaping aerosols and other forms of particulate matter can become trapped within this mucus layer and partake in various aqueous-phase reactions that result in the formation of hydroxyl radicals.^38,39^ To simulate this process, we extracted our vaping particles into an aqueous solution of our spin-trap, BMPO. Figure 2 shows the formation of radical species when vaping aerosols encounter water. This process can be potentially explained by the decomposition of hydroperoxides in aqueous conditions. While on the filter, hydroperoxides could degrade slowly; however, a recent work by Hu et al. shows that water is required for the decomposition of hydroperoxides formed from terpene ozonolysis.^40^ For this to take place, the aged vaping aerosols must undergo dissolution in water, which allows for water-catalyzed and potentially metal-catalyzed decomposition of hydroperoxides. The black spectra indicate the formation of radicals from the interactions of fresh vaping particles with water. The characteristic four-peak spectrum seen in Figure 2A–D is indicative of hydroxyl radicals. The red spectra represent the formation of radicals from aged vaping particles, and the ten-peak spectra are a combination of hydroxyl and alkyl radicals. Once normalized for mass, the aged particles exhibited a greater formation of radicals per microgram of particles, which can be portrayed by the greater areas under the curve (AUC) compared to their fresh counterparts. In Figure 2A, we see radical formation even when no terpenes are present in our vaping aerosols. If these radicals are formed from the decomposition of hydroperoxides, it is plausible that hydroperoxides are being formed directly from the thermal decomposition of PG/VG. It is also possible that certain alkene compounds are being formed from the thermal transformation of PG/VG during vaping and undergoing ozonolysis.^41,58^

The spontaneous aqueous decomposition of hydroperoxides is not likely to fully explain the magnitude of radical formation by these vaping particles. In Figure S4, a 10 mM solution of TBHP, an organic hydroperoxide standard, experiences enhanced radical production in the presence of Fe^2+^. It has previously been reported that various redox-active metals are found in e-cigarette aerosols. These metals originate from the heating element of e-cigarette devices, and every puff results in the release of these metals into vaping emissions.^42^ Many of these metals are known to facilitate Fenton or Fenton-like reactions that induce the decomposition of hydroperoxides and the formation of hydroxyl radicals.^38,39^ Therefore, the abundance of hydroxyl radicals coming from our vaping samples is likely to be influenced by metal–organic interactions and Fenton/Fenton-like reactions that take place under aqueous conditions.

### 3.3 Radical Speciation

**Figure 3:**
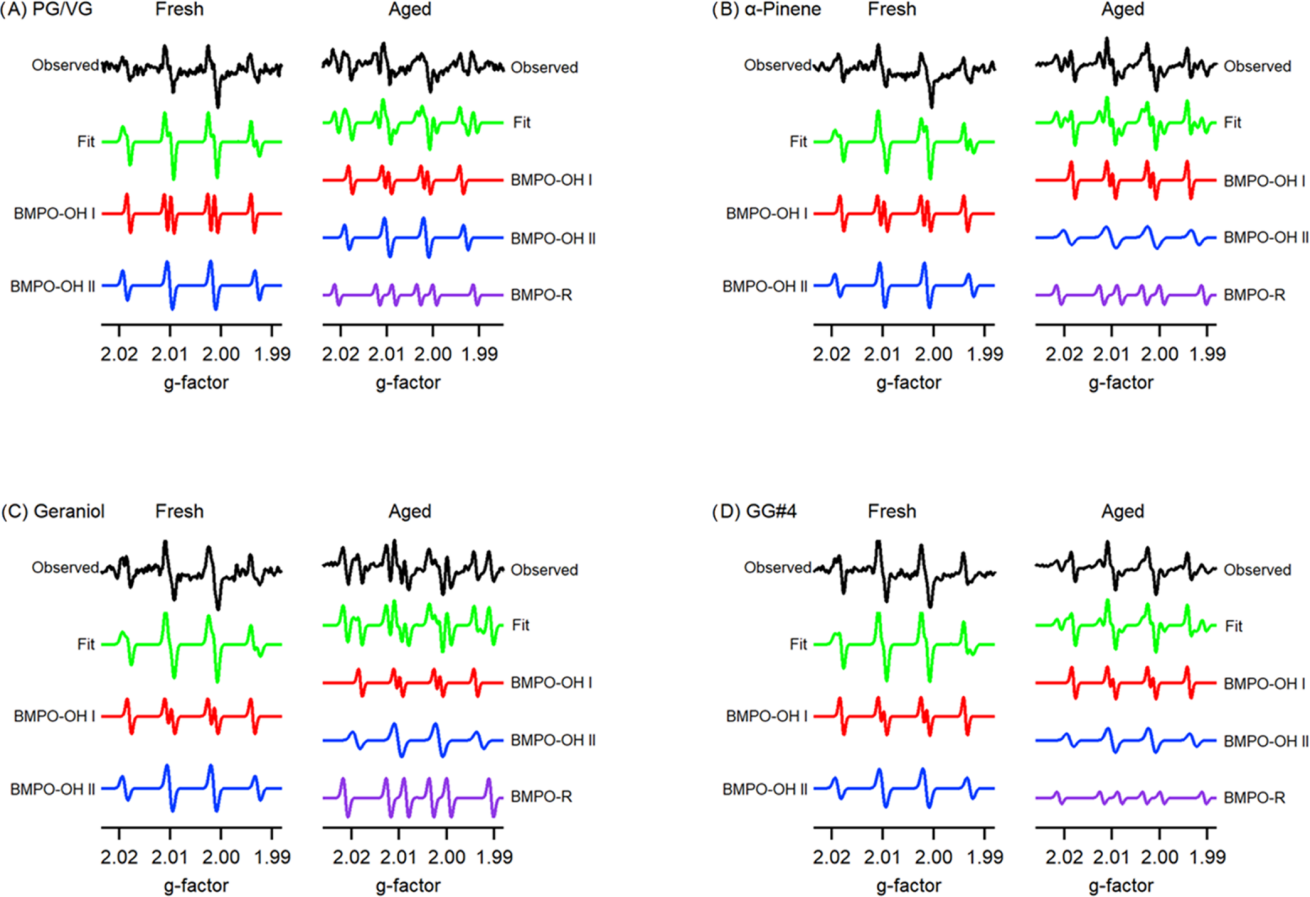
Radical speciation of fresh and aged vaping particles: (A) PG/VG, (B) PG/VG + 3% α-pinene, (C) PG/VG + 3% geraniol, and (D) PG/VG + 3% GG#4. Black spectra represent the observed signal while the green spectra are simulated. BMPO–OH represents the presence of OH radicals while BMPO-R represents the presence of carbon-centered alkyl radicals.

Utilizing the spin-trapping agent, BMPO, we were able to detect the formation of OH radicals when the fresh vaping particles interacted with water (Figure 3). The fresh particles produced spectra with the characteristic four peaks resulting from BMPO–OH hyperfine splitting (Table S1). Both conformers of BMPO–OH were detected in our system, with an even weight distribution between both conformers once simulated. Since it is unlikely for there to be an abundance of hydroperoxides present in our fresh particles, it is evident that there would be an absence of R radicals in the EPR spectra of our fresh particles. However, the detection of BMPO–OH indicates the fresh vaping particle’s ability to produce OH radicals once in solution. Perhaps the heterolytic cleavage of O–O bonds in hydroperoxides favors the formation of OH radicals when hydroperoxide concentrations are relatively low, but other evidence seems to suggest that these signals may be artifacts.^43−45^ BMPO can undergo oxidation by other nonradical oxidants, and a subsequent nucleophilic addition by water can lead to the formation of BMPO–OH.^43^ Therefore, we are not able to conclude whether the fresh vaping particles’ oxidative potential can be attributed to OH radical production or if nonperoxide oxidants and redox-active metals are the major contributors to their oxidative ability.

All of the aged particles resulted in spectra that contained peaks representative of BMPO–OH and BMPO-R hyperfine splitting (Table S1). The aged particles exhibited the ability to form alkyl (R) radicals, which is indicative of hydroperoxide decomposition. Normally, the decomposition of O–O bonds in hydroperoxides results in the formation of OH or alkoxyl (RO) radicals. However, we were not able to detect RO radicals due to their fast rate of decomposition into R radicals.^45^ Therefore, the presence of R radicals in our aged samples can be utilized as an indicator of hydroperoxide formation during the aging process, as well as its decomposition into RO radicals once in aqueous conditions. Interestingly, in Figure 3C, the aged vaping particles containing geraniol showed R radical production with a weight distribution that greatly exceeded OH radical formation. The potentiated ability to produce R radicals in water as well as the rapid O_3_ consumption rate of our geraniol vaping aerosols (Figure S5) highlight geraniol’s reactivity for O_3_ and the efficiency of its ozonolysis. Overall, our EPR results demonstrate that hydroperoxides are likely to be present in the particle phase of our aged vaping aerosols, and their aqueous decomposition into OH and R radicals may act as a mechanism for how they can initiate oxidative damage to exposed tissue.

### 3.4 Molecular Characterization of Particle-Phase Hydroperoxides

**Figure 4:**
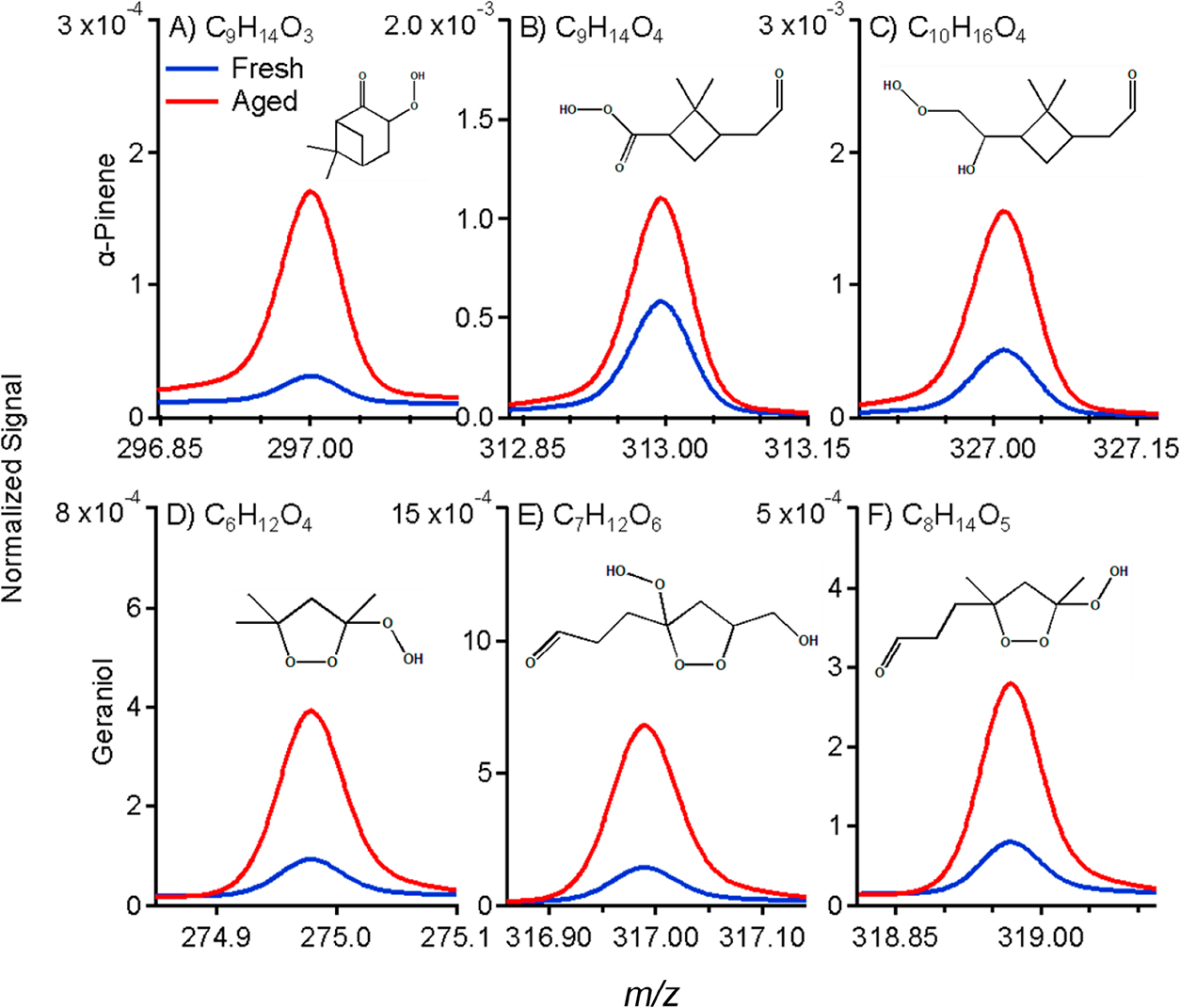
Comparison of the abundance of hydroperoxides found in PG/VG + 3% α-pinene and PG/VG + 3% geraniol vaping particles. Chemical formulas and possible structures of (A) C_9_H_14_O_3_, (B) C_9_H_14_O_4_, and (C) C_10_H_16_O_4_ were detected in our α-pinene vaping particles while (D) C_6_H_12_O_4_, (E) C_7_H_12_O_6_, and (F) C_8_H_14_O_5_ were detected in our geraniol vaping particles by FIGAERO-ToF-CIMS. The formulas of (A), (B), and (C) were compared with reference literature to verify their identities, while the formulas for (D–F) were elucidated from a proposed ozonolysis pathway for geraniol (Figure S6).

Using offline filter analysis in the form of FIGAERO-ToF-CIMS, we were able to detect multiple possible peroxide compounds in our α-pinene vaping particles that have also been previously identified with ESI-MS/MS.^5^ We were also able to detect various peroxide compounds in our geraniol vaping aerosols, whose formulas were determined by a proposed ozonolysis pathway that follows similar mechanisms as α-pinene ozonolysis.^21,57^Figure 4 shows all potential hydroperoxide species that were found in greater abundance in our aged vaping aerosols, as indicated by their greater signal intensity normalized to the PG (C_3_H_8_O_3_I^–^) signal. Our α-pinene vaping particles consisted of hydroperoxides with the formulas C_9_H_14_O_3_, C_9_H_14_O_4_, and C_10_H_16_O_4_. C_10_H_16_O_3_ is a peroxide compound that was also detected in our α-pinene vaping particles, and it was more abundant in the aged particles.

Ozonolysis of geraniol can generate a primary ozonide that can decompose into various possible Criegee intermediates. Subsequent H-shifts followed by Criegee cycloadditions form a multitude of peroxide products (Figure S6) whose chemical formulas were detected with FIGAERO-ToF-CIMS. The geraniol vaping particles contained compounds with the formulas C_6_H_12_O_4_, C_7_H_12_O_6_, and C_8_H_14_O_5._ From our proposed ozonolysis pathway for geraniol, C_8_H_14_O_5_ has two possible peroxide structures; therefore, it is possible that both structures are present within our geraniol vaping particles but cannot be distinguished with CIMS. Consequently, as FIGAERO-ToF-CIMS only predicts molecular formulas but does not provide direct information on functional groups, other methods should be employed in the future to confirm the proposed structures that we elucidated from our ozonolysis pathway.

### 3.5 Quantification of Aerosol-Phase Hydroperoxides

**Figure 5:**
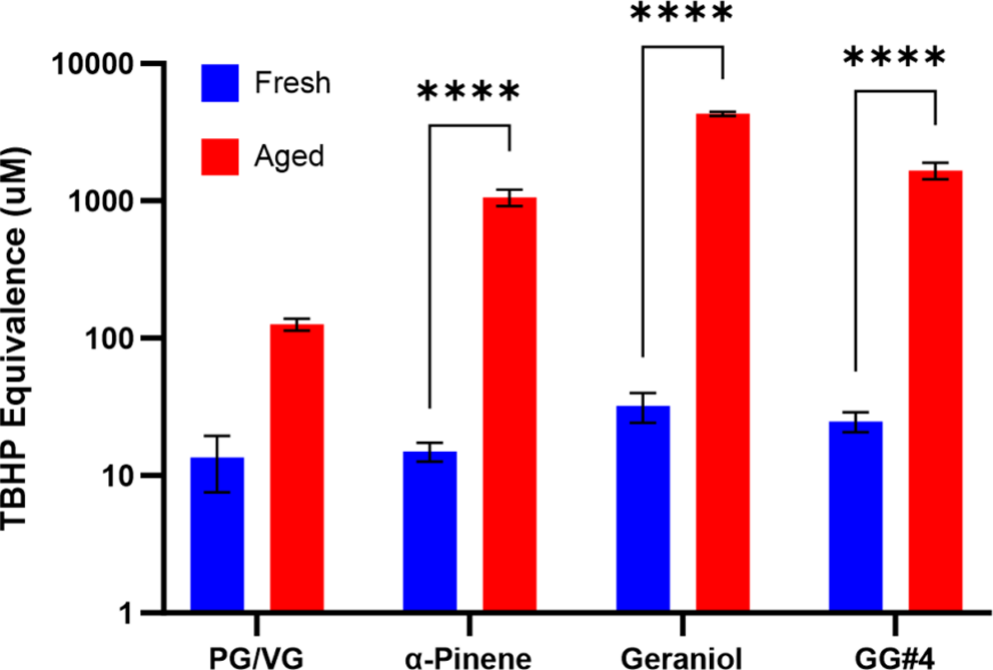
Iodometric quantification of total peroxides present in fresh and aged EC aerosols. Peroxide concentration was quantified based on a TBHP calibration curve, and each column is represented by the mean of three filter replicates (*n* = 3) ± the standard error of the mean (SEM). Two-way ANOVA was used to determine statistical significance and **** indicates *P* < 0.0001.

The quantification of peroxides can be done through the selective oxidation of I^–^ into I_2_ by peroxide compounds. The newly formed I_2_ can react with excess I^–^ to form I_3_^–^, which has a characteristic absorption peak at 350 nm.^46^ Utilizing this process, we were able to see that our aged vaping particles all had significantly more peroxides present, which is evident in Figure 5. The aged filters, apart from PG/VG, had peroxide concentrations that were 2 to 3 orders of magnitude greater than the fresh samples. The results indicate that peroxides were still present in our aged PG/VG samples, which corresponds well with our EPR results and aids in explaining why R radicals are formed when these aged particles interact with water. These results, along with our findings from Figure 4, validate the presence of hydroperoxides in our vaping particles as well as provide evidence that the production of OH and R radicals is mediated by the decomposition of hydroperoxides in our aged vaping particles. We also saw that the aged vaping particles with geraniol contained the most hydroperoxides out of all four groups. The results obtained from the oxidative potential assays (Figure 1) also indicate that aged vaping particles containing geraniol have the greatest oxidative ability out of the four tested e-liquids. Combining the results from both iodometry and oxidative potential assays leads to the conclusion that hydroperoxide abundance is directly correlated with oxidative potential. We can also conclude that the presence of terpene alcohols, such as geraniol, has the potential to be a major contributor to ROS formation during the vaping and aging processes. Lastly, these results also feature the potential toxicity of vaping PG/VG alone and partially explain the cause of clinical symptoms associated with chronic PG/VG exposure, such as coughs and dry throats, that are accompanied by dysregulated expression of genes that maintain the epithelium’s barrier integrity.^47,48^

### 3.6 Gene Expression Analysis

**Figure 6:**
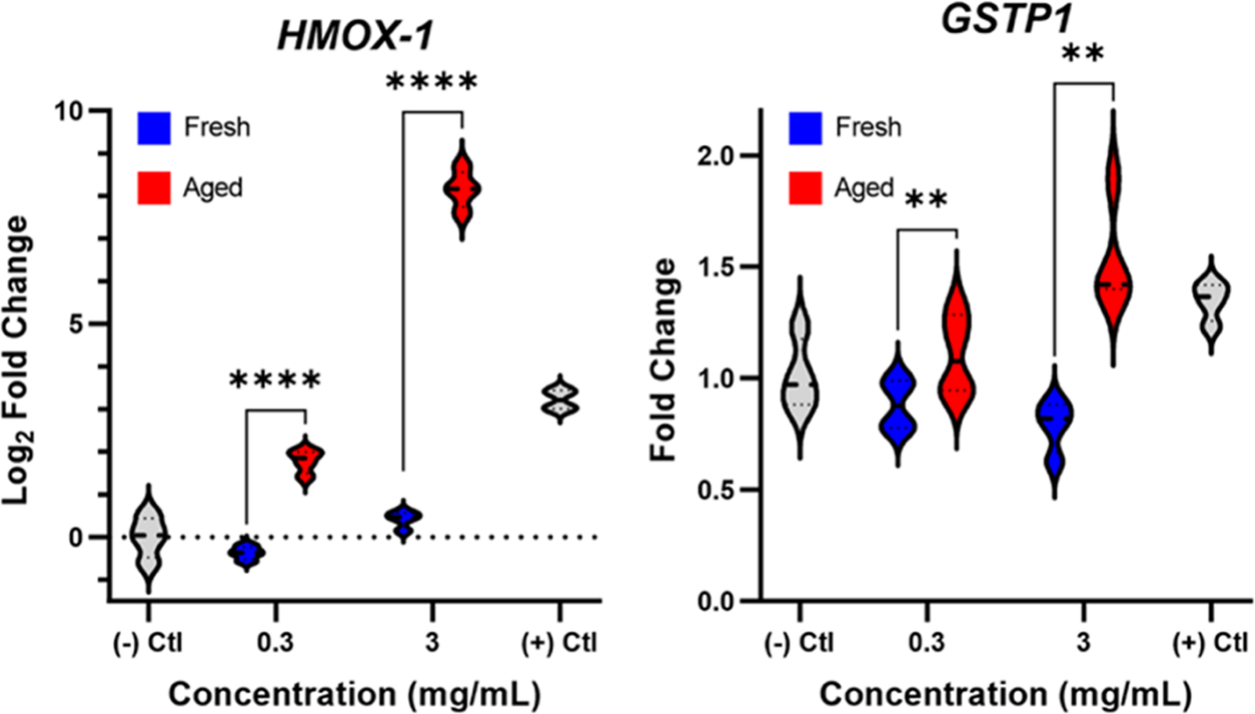
Gene expression of (A) *HMOX-1* and (B) *GSTP1* in BEAS-2B cells after a 6 h exposure to fresh and aged GG#4 vaping particles at concentrations of 0.3 and 3 mg mL^–1^, along with a positive control of 1 mM TBHP. Results are expressed as the median of fold change or log_2_ fold change of 4 biological replicates (*n* = 4) compared to the negative control group that was not exposed along with its interquartile ranges. Two-way ANOVA was used to determine statistical significance. ** indicates *P* < 0.01 and **** indicates *P* < 0.0001.

Exposing BEAS-2B cells to our GG#4 vaping particles led to the upregulation of two oxidative stress biomarkers, *HMOX-1* and *GSTP1*. Previous work has shown that *HMOX-*1 induction is positively associated with ROS exposure, and *HMOX-*1 plays an important cytoprotective role against the development of oxidative stress.^49^*HMOX-*1 is responsible for the catabolism of heme, which leads to the formation of bilirubin and carbon monoxide, potent antioxidant and antiapoptotic agents, respectively.^49^Figure 6 shows that both 0.3 and 3 mg mL^–1^ of our aged vaping particles were able to induce significant dose-dependent increases in the expression of *HMOX-1* mRNA. However, the fresh vaping particles did not exhibit any effect on *HMOX-1* transcription. Although the fresh vaping particles did not undergo any significant aging and ozonolysis, thermal degradation and auto-oxidation of PG/VG and our terpene mixture are likely to have formed various highly oxidized species that act as ROS or facilitate the production of intracellular ROS.^50^ It is a possibility that unreacted terpenes present within the fresh vaping particles are acting as antioxidants^51^ and effectively dampening the oxidative potential of the fresh vaping particles.

Like other glutathione S-transferases, *GSTP1* is a crucial regulator of intracellular ROS levels.^52,59^ Exposure to our aged vaping particles induced the upregulation of *GSTP1*, indicating a rise in intracellular ROS. The induction of *GSTP1* expression is a protective mechanism against oxidative stress, but this antioxidant system can become overwhelmed by excess ROS as cellular glutathione starts to deplete.^52^ The expression of these two biomarkers was seen to be dose-dependent when BEAS-2B cells were exposed to the aged emissions, indicating a direct relationship between hydroperoxide exposure and oxidative stress within human bronchial epithelial cells.

## 4 Conclusions

The use of e-cigarettes and the production of their aerosols are commonly polluting indoor areas where these vaping emissions are subject to indoor oxidants such as O_3_. Indoor O_3_ mainly originates from outdoor tropospheric O_3_ that has gradually diffused into the indoor atmosphere.^53^ O_3_ can react with common monoterpenes found in vaping emissions^21^ to form various products that are potentially toxic to people who experience secondhand exposure. When simulating the oxidative conditions of an indoor environment within a controlled smog chamber, we found that vaping emissions that undergo O_3_-mediated aging form more oxidative particles than the ones that were originally emitted from the e-cigarette. All four of our tested e-liquids produced aged particles that had greater oxidative potential than the non-aged forms, with the aged geraniol vaping particles containing the most ROS. EPR spin-trapping analysis showed the formation of hydroxyl and alkyl radicals once these aged vaping particles interacted with water. The aged samples consistently produced more radicals, detected as BMPO adducts, than what the fresh particles could produce. Using FIGAERO-ToF-CIMS, we were able to detect the presence of various hydroperoxides and peroxy-acids that are known products of α-pinene and geraniol ozonolysis. These compounds were detected in higher abundance in the aged particles, and the abundance of particle-phase hydroperoxides was quantified utilizing iodometry. Unsurprisingly, we determined that there were significantly more hydroperoxides that were present in the aged vaping particles, and the aged geraniol vaping particles contained the most hydroperoxides out of the four groups.

All these findings point out the potential harms of exposure to aged e-cigarette emissions. Although exposure to low concentrations of these vaping emissions for a short period of time is not likely to induce any respiratory dysfunction,^1^ it is not an unrealistic scenario for one to be exposed to indoor areas where habitual e-cigarette users produce massive amounts of vaping emissions.^8^ Consequently, chronic exposure to aged vaping particles is likely to cause significant respiratory damage and oxidative stress. In this study, we proposed that the inhalation of our aged vaping particles will lead to their interaction with the fluid that is lining the respiratory tract. Particle-phase hydroperoxides formed from the aging process will decompose into hydroxyl and alkyl radicals that may directly induce oxidative damage to the respiratory epithelium.^38,39^ Unreacted hydroperoxides can interact with epithelial cell membranes, facilitating lipid peroxidation and eventually causing cell death.^54^ Diffusion and transport of these hydroperoxides into epithelial cells and the systemic circulation have the potential to cause more intracellular damage and systemic toxicity, respectively.^55,56^ Exposing human bronchial epithelial cells to our aged vaping particles resulted in the upregulation of oxidative stress biomarkers, *HMOX-1* and *GSTP1*, which suggests that the aging process produced a significant amount of ROS that can induce oxidative stress in our cell culture model.

Lastly, the ability for vaping emissions to linger in the air and stay suspended for long periods of time^8−10^ makes ambient aging a significant transformation process that should be studied further. However, there are ways to mitigate this process and reduce the risk of exposure to aged vaping emissions. Indoor areas with poor ventilation are likely to have greater concentrations of VOCs and particulate matter;^11^ therefore, proper ventilation in homes and other living spaces should be in place to minimize the accumulation of vaping emissions in confined areas. In places with high ground-level O_3_, it is difficult to control the concentration of indoor O_3_. However, various air filtration methods, normally used for industrial and research purposes, have been employed to reduce indoor O_3_ concentrations in the residential setting.^53^ Overall, these methods may be effective at reducing the risk of involuntary secondhand exposure, but there will always be some level of risk if vaping emissions are present.

## Supporting Information Available

Hyperfine splitting constants of our BMPO adducts obtained from simulated spectra (Table S1); particle mass concentrations throughout the duration of our chamber studies (Figure S1); TBHP calibration curve used for iodometric quantification of particle-phase peroxides (Figure S2); particle number concentration by aerodynamic diameter (Figure S3); EPR spectra of 10 mM TBHP in an aqueous solution of 20 mM BMPO (Figure S4); ozone consumption during the aging process (Figure S5); geraniol ozonolysis pathway (Figure S6) (PDF)

## Abbreviations

PG: propylene glycol
VG: vegetable glycerin
ROS: reactive oxygen species
SOA: secondary organic aerosol
FIGAERO: filter inlet for gases and aerosols
ToF: time of flight
CIMS: chemical ionization mass spectrometry
EPR: electron paramagnetic resonance
COPD: chronic obstructive pulmonary disease
BMPO: 5-*tert*-butoxycarbonyl-5-methyl-1-pyrroline-*N*-oxide
TBHP: *tert*-butyl hydroperoxide
ESI: electrospray ionization
MS/MS: tandem mass spectrometry
VOC: volatile organic compound
