# Supplementary material for: Ozonolysis of Terpene Flavor Additives in Vaping Emissions: Elevated Production of Reactive Oxygen Species and Oxidative Stress
Source: Chem Res Toxicol. 2024 May 22;37(6):981–90. doi: 10.1021/acs.chemrestox.4c00051 (PMC11187633; doi:10.1021/acs.chemrestox.4c00051)
Supplement: Supplementary file 1 — tx4c00051_si_001.pdf [file tx4c00051_si_001.pdf]

*Supporting Information*

# Ozonolysis of terpene flavor additives in vaping emissions: Elevated production of reactive oxygen species and oxidative stress

Wonsik Woo,[1] Linhui Tian,[2] Michael Lum,[2] Alexa Canchola,[1] Kunpeng Chen[2], Ying-Hsuan Lin,[1,2*]

[1]Environmental Toxicology Graduate Program, University of California, Riverside, California, 92521, United States

[2]Department of Environmental Sciences, University of California, Riverside, California, 92521, United States

*Corresponding Author

Email: ying-hsuan.lin@ucr.edu

**Content:**

Number of pages: 8

Number of tables: 1

Number of figures: 6

**Table S1.** Hyperfine splitting constants (HFS) of our BMPO adducts obtained from simulated spectra presented as their mean ± standard deviation.

| BMPO adducts | HFS (G) | Fresh | Aged |
|---|---|---|---|
| BMPO-OH I | $\alpha_N$ | 39.86 ± 0.15 | 39.78 ± 0.08 |
| | $\alpha_{H\beta}$ | 34.57 ± 0.27 | 34.6 ± 0.4 |
| | $\alpha_{H\gamma}$ | 1.02 ± 0.11 | 1.01 ± 0.07 |
| BMPO-OH II | $\alpha_N$ | 40.57 ± 0.38 | 40.27 ± 0.43 |
| | $\alpha_{H\beta}$ | 41.33 ± 0.45 | 42.49 ± 1.45 |
| | $\alpha_{H\gamma}$ | 2.02 ± 0.008 | 2.04 ± 0.13 |
| BMPO-R | $\alpha_N$ | - | 42.02 ± 0.2 |
| | $\alpha_{H\beta}$ | - | 59.15 ± 0.63 |

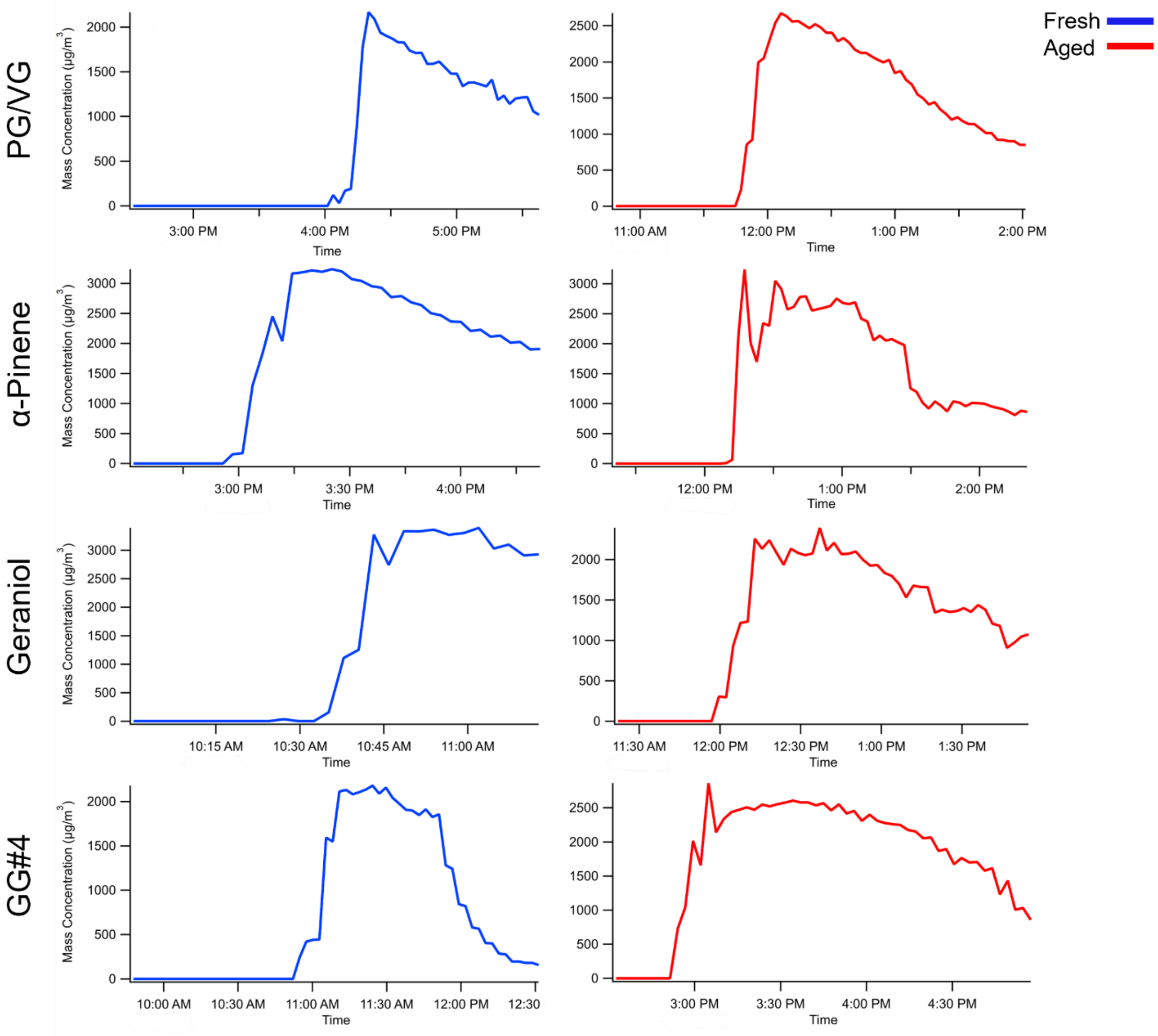


**Figure S1.** Particle mass concentrations throughout the duration of our chamber studies.

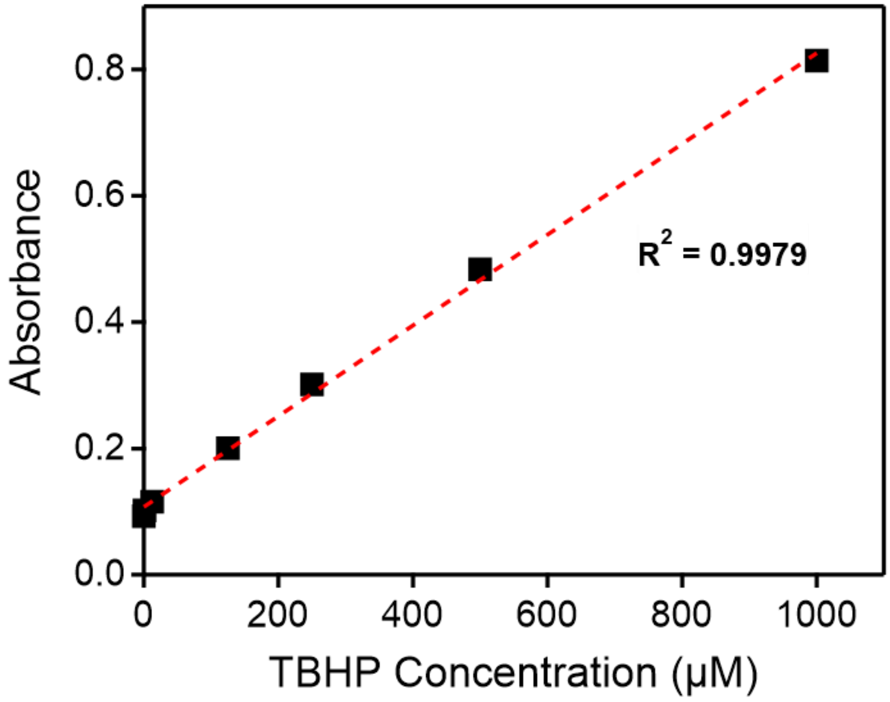


**Figure S2.** TBHP calibration curve used for iodometric quantification of particle-phase hydroperoxides.

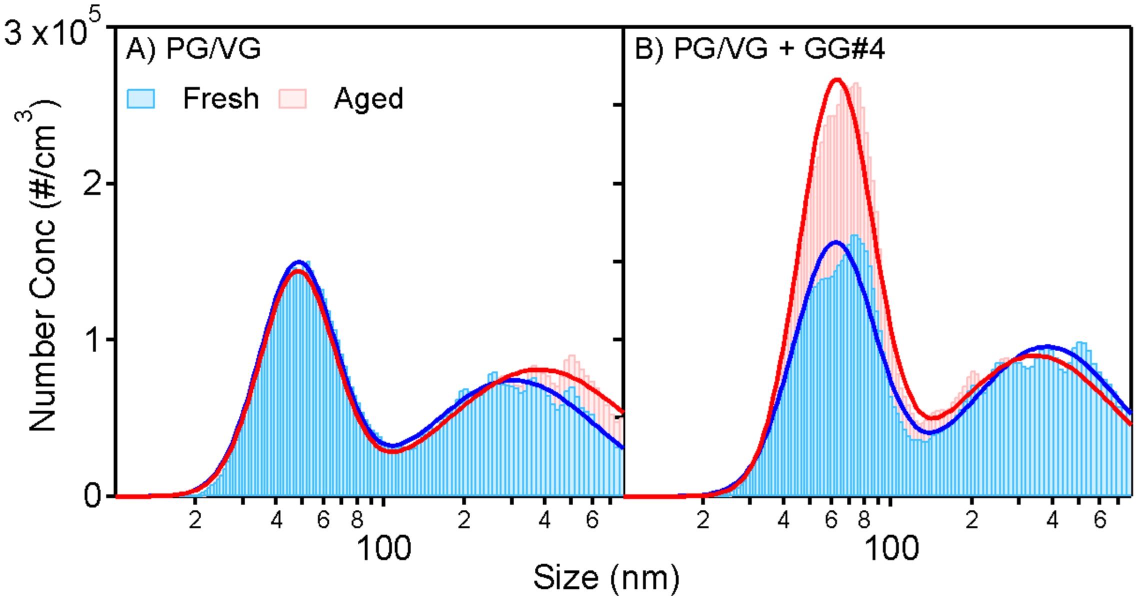


**Figure S3.** Particle number concentration by aerodynamic diameter.

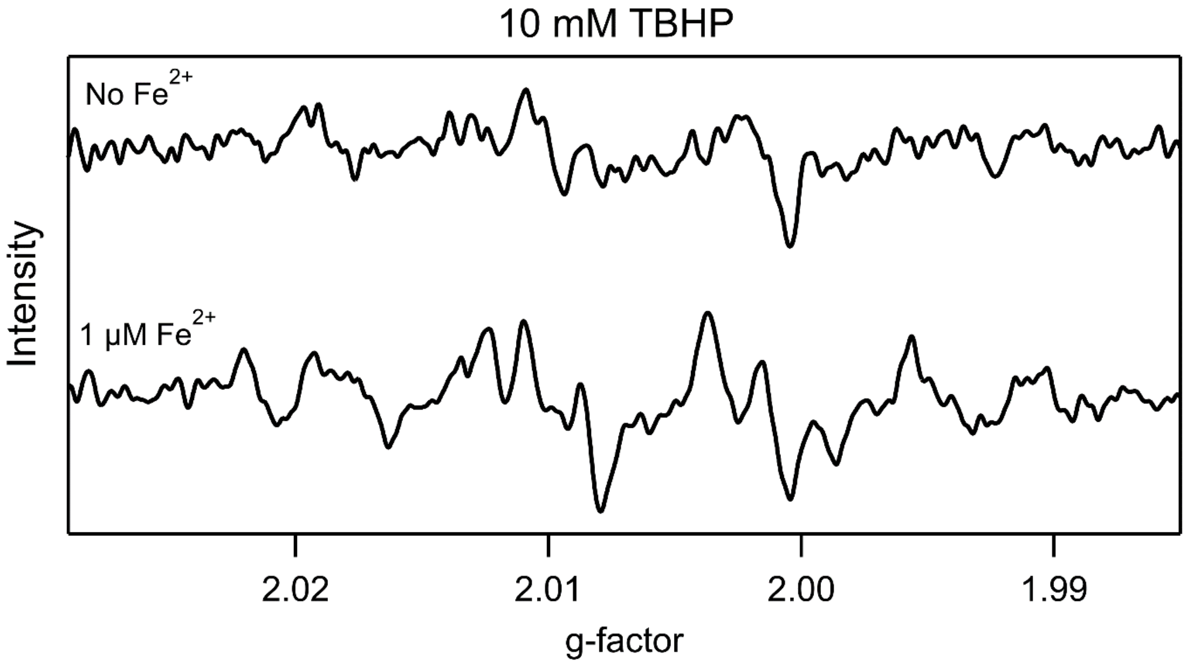


**Figure S4.** EPR spectra of 10 mM TBHP in an aqueous solution of 20 mM BMPO in the presence and absence of 1 μM $Fe^{2+}$.

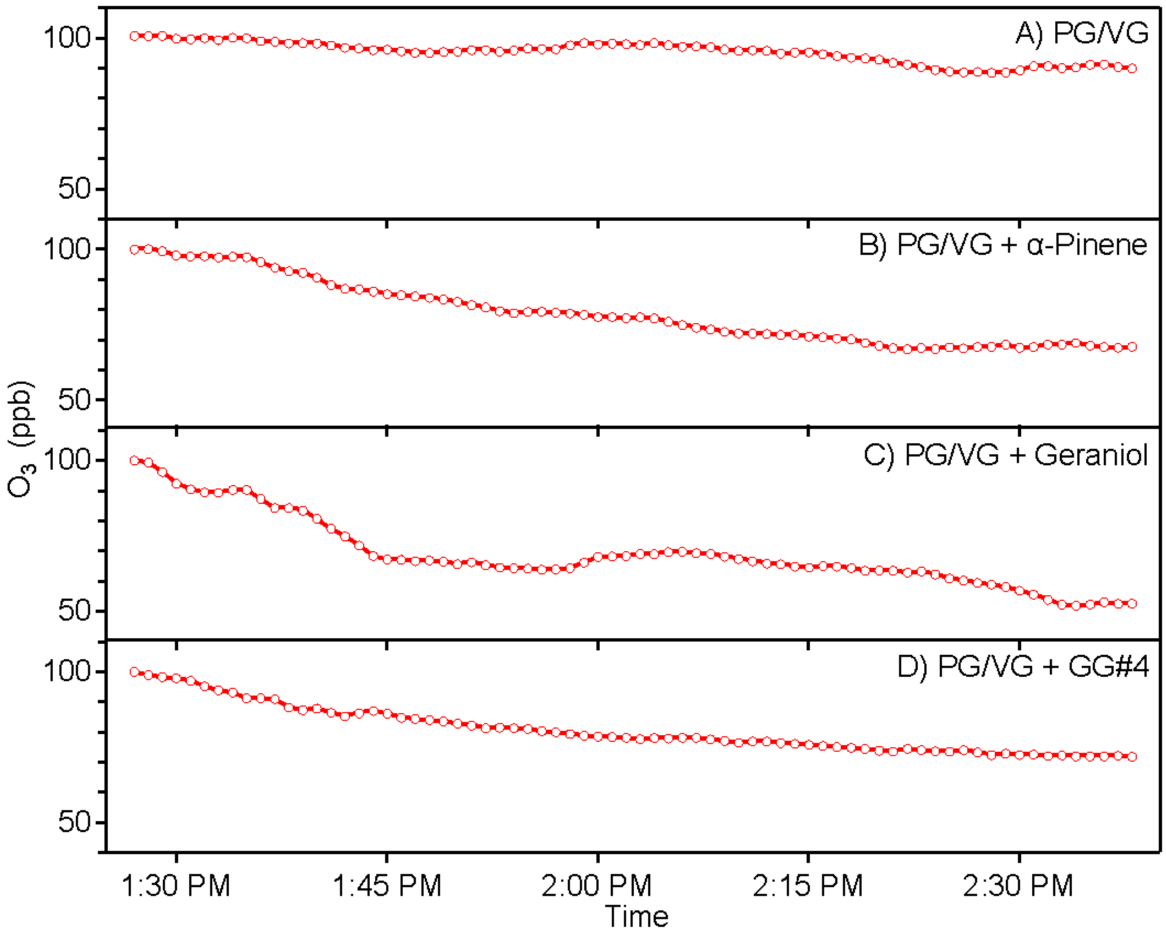


**Figure S5.** Ozone consumption during the chemical aging process.

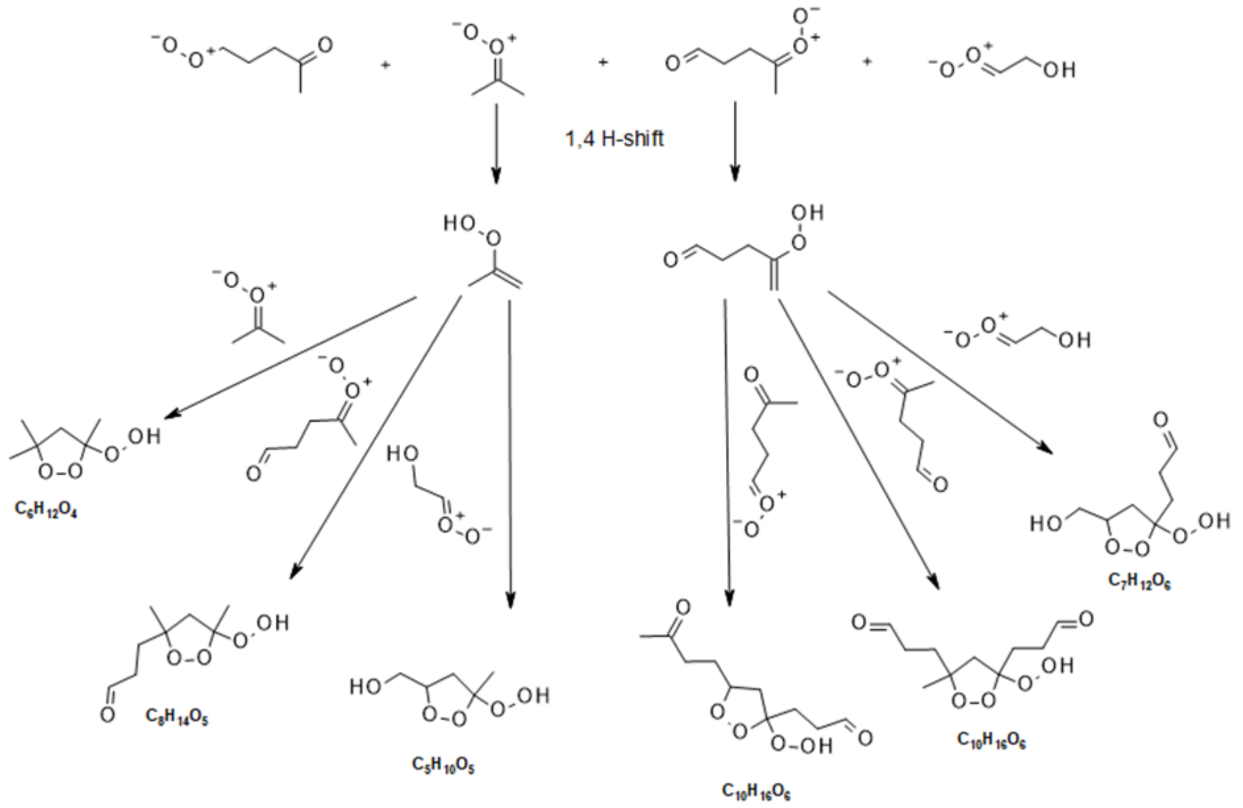


**Figure S6.** Proposed pathways for ozonolysis of geraniol leading to the formation of organic hydroperoxides.
